# Conventional ovarian stimulation vs. delayed single dose corifollitropin alfa ovarian stimulation in oocyte donors: a prospective randomized study. Tail trial

**DOI:** 10.3389/frph.2023.1239175

**Published:** 2023-10-30

**Authors:** Carlos Alberto Alvarado Franco, Andrea Bernabeu García, Jordi Suñol Sala, Jaime Guerrero Villena, Sonia Albero Amorós, Joaquin Llacer, Ricardo Antonio Delgado Navas, José A. Ortiz, Anna Pitas, Juan Carlos Castillo Farfan, Rafael Bernabeu Pérez

**Affiliations:** ^1^ Department of Gynecology, Instituto Bernabeu, Cartagena, Spain; ^2^ Co-direction Department, Instituto Bernabeu, Alicante, Spain; ^3^ Department of Gynecology, Instituto Bernabeu, Palma de Mallorca, Spain; ^4^ Department of Embryologist, Oocyte Donation, Instituto Bernabeu, Alicante, Spain; ^5^ Department of Gynecology, Accuna, Alicante, Spain; ^6^ Department of Gynecology, Instituto Bernabeu, Alicante, Spain; ^7^ Department of Genetic, BIOTECH, Alicante, Spain; ^8^ Clinical Trials Project Manager, Instituto Bernabeu, Alicante, Spain; ^9^ Community Medicine and Reproductive Health Chair, Miguel Hernandez University, Elche, Spain; ^10^ Management Department, Instituto Bernabeu, Alicante, Spain

**Keywords:** corifollitropin alfa, controlled ovarian stimulation, recombinant FSH, MII oocytes, cumulus-oocyte complexes

## Abstract

The present study compares two protocols for ovarian controlled stimulation in terms of number of cumulus-oocyte complexes and metaphase II oocytes. We employed a single injection of 150mcg of corifollitropin alfa after a 7-day oral contraceptive pill-free interval for TAIL group and a conventional administration of corifollitropin alfa after a 5-day OCP-free interval with additional rFSH from 8th of ovarian controlled stimulation. Prospective, randomized, comparative, non-inferiority, opened and controlled trial carried out in 180 oocyte donors 31 were excluded, 81 were randomized to the control group and 68 to the TAIL group. No differences were found in the number of follicles larger than 14 and 17 mm at triggering day. However, a lower number of cumulus-oocyte complexes and metaphase II oocytes were obtained in TAIL group compared to the control group, expressed as median (interquartile range): 10.5 (5.5–19) vs. 14 [11–21] and 9 (4–13) vs. 12 (9–17) respectively. Additionally, the incidence of failed retrieval or metaphase II oocytes = 0 was higher in TAIL group 7(10.3%) vs. 1(1.2%) *p* = 0.024. The use of a single injection of corifollitropin alfa after a 7-day oral contraceptive pill-free interval in oocyte donors resulted in a lower number of cumulus-oocyte complexes and metaphase II oocytes. No additional rFSH was administered in this group.

**Clinical Trial Registration:** https://www.clinicaltrialsregister.eu/ctr-search/trial/2019-001343-44/results.

## 1. Introduction

One of the most critical factors influencing the outcomes of assisted reproduction treatments (ART) is advanced maternal age (1, 2). Due to changing societal trends, many women are now choosing motherhood at an age above 40 years, which is why a significant number of them require ART, particularly oocyte donation programs (2, 3).

Oocyte donation programs now constitute a substantial portion of current ART cycles worldwide. It is of utmost importance to optimize ovarian stimulation protocols while ensuring safety and convenience, especially for the population of young donors.

Unlike current treatment regimens that require daily injections of exogenous gonadotrophins to maintain adequate levels of FSH during ovarian controlled stimulation (COS) due to their short elimination half-life and rapid metabolic clearance, the new molecule with sustained follicle-stimulating activity, long-acting rFSH—Corifollitropin alfa (CFA), provides a plasma half-life of approximately 65 h. This extended duration of action allows it to replace the first seven injections of standard daily gonadotropins (4). Because of its low elimination rate, some activity persists beyond those initial 7 days (5). We can anticipate a gradual reduction in its effect beyond the first seven days of medication administration, which is likely sufficient to complete the ovarian stimulation process. In oocyte donation programs, the use of CFA offers the advantages of patient-friendliness and convenience while also having the potential to reduce administration errors, provided that safety and efficiency are first demonstrated.

Oral contraceptive pills (OCP) are commonly used in oocyte donation cycles, as they can facilitate scheduling and synchronization with recipients without negatively affecting the outcomes. Importantly, pre-treatment with OCP in IVF cycles does not appear to compromise oocyte quality (6).

In a recent study by Pérez-Calvo *et al*. (7), a comparison was made between two OCP-free intervals: 5-day and 7-day, both using Corifollitropin alfa (CFA) in an antagonist protocol. The findings suggested that extending the OCP-free interval to 7 days significantly reduces the total dose of gonadotrophins, shortens the duration of stimulation, and decreases the total number of injections. This protocol involved additional daily FSH doses after day 7 of stimulation when needed and was conducted with a limited cohort of donors in a prospective design.

The objective of the present study is to assess the efficiency of ovarian stimulation in oocyte donation cycles. We compare two protocols: one utilizing a single injection of CFA 7 days after discontinuing contraceptive pills without additional rFSH (referred to as the TAIL protocol), and the other involving CFA administration 5 days after stopping contraceptive pills with additional rFSH starting from the 8th day of Controlled Ovarian Stimulation (COS). Our focus is on laboratory outcomes, specifically oocyte yield and the production of metaphase II oocytes. This study is conducted on a larger scale as a randomized clinical trial.

## 2. Material and methods

### 2.1. Patient selection

This single-center prospective randomized study included oocyte donation cycles performed at ACCUNA fertility center in Alicante - Spain, between November 2019 and April 2021.

All donors included in the study were healthy women 18–32 years, with a body mass index between 18 and 29 kg/m^2^, an antral follicle count (AFC) > 12, both ovaries present, with regular menstrual cycles and recruited according to the clinical and legal requirements of the Spanish Act for Assisted Human Reproduction: Reproductive Act (RD 9/2014) which includes: a psychological interview, gynecological examination and a rigorous screening for infectious diseases and genetic abnormalities. Donors signed the corresponding informed consent form during enrollment.

The exclusion criteria included: endometriosis, AFC > 20, polycystic ovarian syndrome (PCOS) and concurrent participation in another study.

### 2.2. Stimulation protocols

**Figure 1:**
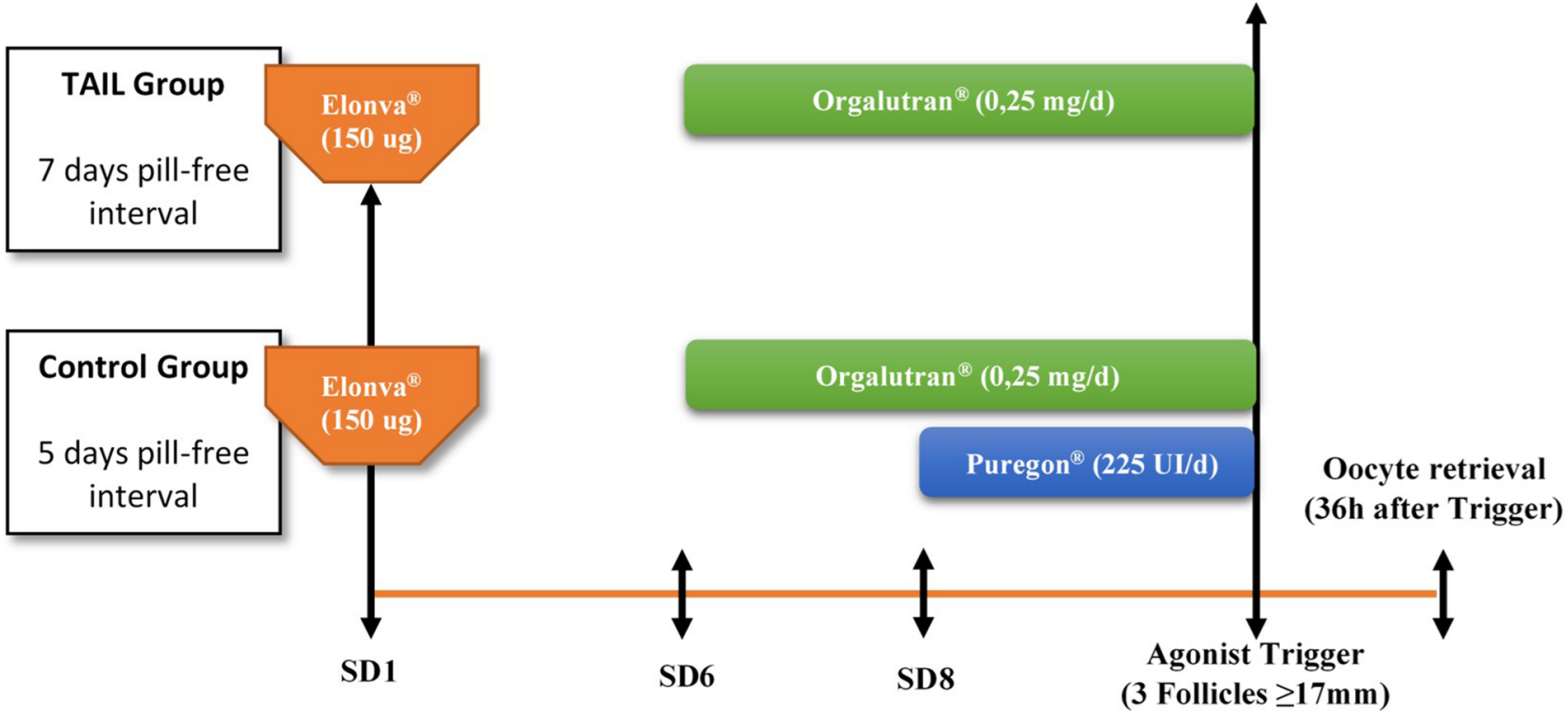
Stimulation protocol. SD, Stimulation Day.

All donors were enrolled prospectively and started with OCP (ethinylestradiol 30 ug and desogestrel 150 ugs; Microdiol®, Oragnon Salud, Spain) for 14–34 days. While on contraceptives, donors were randomized to one of the two study arms by simple randomization: TAIL group, only a single dose of 150 ugs of CFA (Elonva®, MSD, Spain) was used for ovarian stimulation administrated 7 days after OCP cessation, whereas in the control group, a single dose of 150 ugs of CFA plus additional 225 UI of rFSH (Puregon®, MSD, Spain) supplementation from 8th day of COS (if required) were administrated 5 days after OCP discontinuation. A daily administration of 0.25 mg GnRH antagonist ganirelix (Orgalutran®, MSD, Spain) was initiated on stimulation day 6 in both groups to prevent a premature LH surge until the trigger day. A bolus of a GnRH agonist (Triptorelin 0.2 mg, Decapeptyl®, Ipsen Pharma, Barcelona, Spain) was used for final oocyte maturation when at least 3 follicles were ≥17 mm in diameter. Oocyte aspiration was performed 36 h after GnRH agonist injection by transvaginal ultrasound-guided needle-aspiration. (Figure 1).

Five follow-up controls during the stimulation process were scheduled: on the first day of COS (visit 1), fifth day of COS (visit 2), eighth day of COS (visit 3), at the day of agonist administration (visit 4) and the day of oocyte retrieval (visit 5).

### 2.3. Outcomes

The primary outcome was the number of cumulus-oocyte complexes (COCs) collected and number of metaphase II (MII) oocytes. Secondary objectives included: number of follicles ≥14 and 17 mm on the day of triggering, additional doses of recombinant FSH, antagonist injections (days), duration of ovarian stimulation (days) and endocrine profile. Other outcomes included the overall cost of gonadotropin consumption.

### 2.4. Randomization and allocation of patients

Once patient eligibility was confirmed, and informed consent was obtained, donors who were currently using contraceptives were randomly assigned to one of the two study arms. Randomization was achieved through a computer-generated list, ensuring a 1:1 allocation ratio. This randomization list was generated using the statistical program SAS® [PLAN procedure, Copyright(c) 2002–2012 by SAS Institute Inc., Cary, NC, USA], with the aim of providing an equal probability of assignment to both treatments. Importantly, the investigators had no access to this list.

A nurse responsible for the allocation process placed each treatment assignment in a sealed, opaque envelope, and these envelopes were retrieved consecutively at the time of randomization. It's worth noting that this study was not conducted in a blinded fashion.

### 2.5. Statistics

Our sample size calculation was based on data from a previous publication by Pérez - Calvo et al. 2017. Assuming that a mean of 13 oocytes is expected in the control group and accepting an alpha risk of 0.05 and a beta risk of 0.10 in a one-sided contrast (statistical power of 90%), a sample size of 150 patients (75 in each study group) is required to detect a minimum difference of 3 oocytes with a standard deviation of 5.2 points using a margin of non-inferiority of 0.5 (8). Estimating a drop-out loss rate of 15%, a sample size of 180 donors was required (90 per group).

The descriptive statistical methods used in this study will depend on the type of the variable analyzed. In the case of qualitative variables, the following descriptive statistics will be obtained: frequency and percentage. For quantitative variables, descriptive analysis was done using the mean, median and standard deviation.

For the univariate statistical analysis of qualitative variables, the Chi - square test or Fisher's exact test will be used. For evaluation of normal distributions, the Shapiro—Wilk's test was performed. Depending on whether the variable has a normal distribution, the comparison between means was carried out using Student's *t* test or Wilcoxon rank sum test. Values of *p* < 0.05 will be considered statistically significant.

The statistical analysis was performed with R Statistical Software, version 4.0.3 and the Software Statistical Product and Service Solutions, version 20.0 (SPSS, Chicago, IL, EE.UU.).

## 3. Results

**Figure 2:**
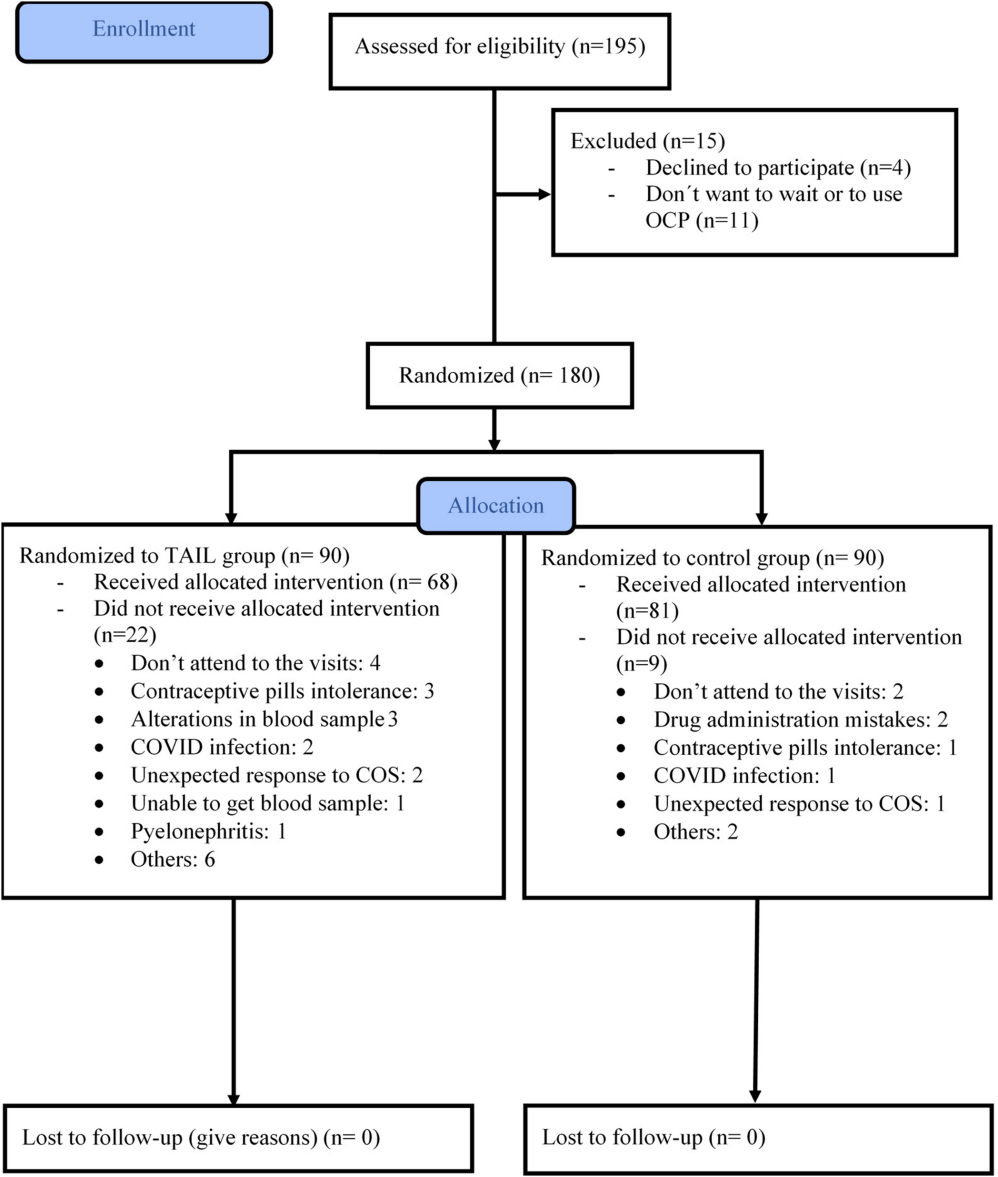
Oocyte donor's enrollment.

Out of a total of 180 oocyte donors, 90 were assigned to the TAIL group, and the remaining 90 were allocated to the control group. Baseline characteristics were comparable between the two groups (see Table 1). Subsequently, 31 donors were excluded from the per protocol analysis, as detailed in Figure 2. Ultimately, our analysis included 149 oocyte donors who successfully completed the treatment protocol: 81 in the control group and 68 in the TAIL group.

**Table 1:** Main characteristics of egg donors receiving COS under the conventional protocol with CFA (control group) vs. delayed single dose CFA (TAIL group).

	Group	Median	IQR^a^
Age (years)	Control	24.97	21.71–28.62
	TAIL	25.05	22.09–27.48
Smoker (%)	Control	51.7	
	TAIL	36.7	
BMI^a^	Control	22.23	21.05–24.74
	TAIL	22.0	20.07–23.74
Previous ovarian stimulations	Control	2	1–5
	TAIL	2	1–3
Oocytes retrieved in previous stimulations	Control	12	10–16
	TAIL	12	8–16
AMH^b^	Control	21.16	15.13–32.31
	TAIL	21.27	12.96–39.91
AFC^c^	Control	15	14–17
	TAIL	16	14–18

^a^ IQR, Interquartile range.

^b^ BMI, Body mass index (Kg/m^2^).

^c^ AMH, anti-Mullerian hormone, pmol/L.

^d^ AFC, Antral follicle count.

### 3.1. Primary outcome measure

In the TAIL group, there was a significantly lower number of COCs) [10.5 (5.5–19)] compared to the control group [14 (11–21)], with a *p*-value of <0.01. Additionally, the TAIL group yielded fewer MII oocytes [9 (4–13)] compared to the control group [12 (9–17)], with a *p*-value of <0.001 (Table 2).

**Table 2:** Overall outcome of COS comparison between TAIL and control group.

	TAIL	Control	*p*-value***
Number of patients	68	81	
Number of COC*	10.5 (5.5–19)	14 (11 -21)	0.0031^a^
Number of MII*	9 (4 -13)	12 (9–17)	<0.001^a^
Total cost of gonadotropins (€)*	609.77	812.27 (756.02–913.52)	<0.0001^a^
MII/€*	0.01 (0.01–0.02)	0.01 (0.01–0.02)	0.65^a^
Categorized “Number of MII”	47 (69.11%)	76 (93.83%)	0.00014^b^
(n MII ≥ 6)**			
Categorized “Number of MII”	21 (30.88%)	5 (6.17%)	
(n MII < 6)**			
Categorized “Number of MII”	61 (89.7%)	80 (98.8%)	0.024^b^
(n MII > 0)**			
Categorized “Number of MII”	7 (10.3%)	1 (1.2%)	
(n MII = 0)**			
Number of MII (oocyte retrieval >0) *	10 (6–14) (*n* = 61)	12 (9–17.25) (*n* = 80)	0.006^a^

COC, Cumulus-oocyte complexes; MII, Metaphase II oocytes; €, Euros.

^a^ Wilcoxon rank sum test.

^b^ Fisher's exact test.

* Values are expressed as Median (Interquartile range).

** Values are expressed as number (Percentage).

*** *p* < 0.05 is significant.

### 3.2. Secondary outcomes and other efficacy endpoints results

The incidence of MII = 0 and a low number of MII oocytes (<6 MII) was notably higher in the TAIL group compared to the control group, with percentages of 10.3% vs. 1.2% and 30.88% vs. 6.17% respectively. Out of 149 oocyte retrievals, 8 cases (5.37%) resulted in MII = 0 at retrieval, with 7 (10.3%) occurring in the TAIL group and 1 (1.2%) in the control group. Detailed laboratory outcomes are presented in Table 2.

The TAIL group had a shorter duration of ovarian stimulation until trigger (8.66 ± 1.36 days) compared to the control group (9.2 ± 1.45 days), with a significant difference (*p* = 0.01), values expressed as Mean ± SD. Additionally, the TAIL group required fewer days of antagonist injections 5 (5–6) days compared to the control group 6 (5–6) days, with a highly significant difference (*p* < 0.01). In contrast, the control group required an additional 3 (2–3) days of rFSH administration, values expressed as median (Interquartile range: IQR). (Table 3).

**Table 3:** Overall outcome of COS comparison between control and TAIL group.

	TAIL	Control	*p*-value**
Number of patients	68	81	
Duration of COS (days)*	9 (8–9)	9 (8–9)	0.01^a^
Use of rFSH (Puregon®) (days)*	0	3 (2–3)	-
Use of antagonist (Orgalutran®) (days)*	5 (5–6)	6 (5–6)	0.0062^a^
Number of follicles larger than 14 mm on the day of the agonist*	17 (13–22.75)	16.5 (13–21)	0.85^a^
Number of follicles larger than 17 mm on the day of the agonist*	9 (7–12)	9.5 (7.75–12)	0.29^a^

COS, Controlled ovarian stimulation (days); rFSH, Recombinant follicle stimulating hormone, IU/L.

^a^ Wilcoxon rank sum test.

* Values are expressed as Median (IQR).

** *p* < 0.05 is significant.

**Figure 3:**
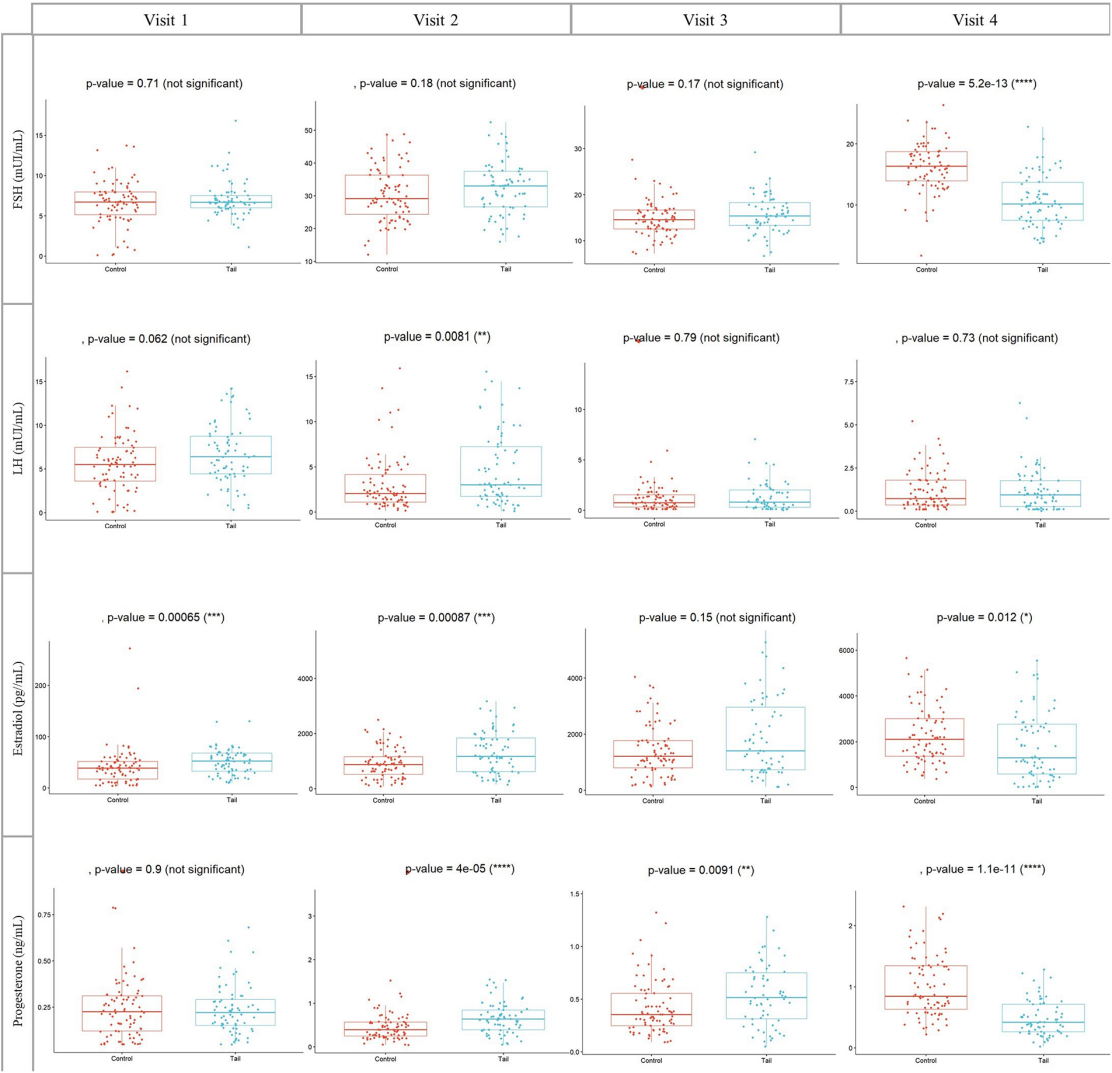
Concentrations of FSH, LH, estradiol and progesterone during COS in both groups. Visit 1: at the first day of COS, visit 2: at the fifth day of COS, visit 3: at the eighth day of COS and visit 4: at the day of GnRH agonist trigger. COS, Controlled ovarian stimulation (days); FSH, Follicle stimulating hormone, IU/l. LH, Luteinizing hormone; IU/l. Wilcoxon rank sum test, *p* < 0.05 is significant.

Hormonal profiles of both groups during Controlled Ovarian Stimulation (COS) are depicted in Figure 3, with values expressed as Median (Interquartile range: IQR). Notably, the TAIL group exhibited lower levels of FSH at the triggering day (visit 4) [10.19 (7.50–13.69) vs. 16.36 (13.93–18.71), *p* < 0.01].

Regarding LH, E2 and Progesterone levels, significant differences were also observed. In the TAIL group, LH levels at visit 2 were higher [3.02 (1.73–7.22) vs. 2.04 (1.10–4.14), *p* = 0.008]. E2 levels at visit 1 [52.34 (32.60–67.73) vs. 38.47 (17.16–51.60), *p* < 0.001] and visit 2 [1175 (624.20–1835) vs. 880 (519.95–1160), *p* < 0.001] were higher, whereas at visit 4 [1296.50 (584.40–2764.25) vs. 2107.50 (1363–2997), *p* = 0.012], E2 levels were lower.

Additionally, PG levels at visit 2 [0.64 (0.39–0.84) vs. 0.39 (0.24–0.57), *p* < 0.001] and visit 3 [0.52 (0.31–0.75) vs. 0.36 (0.25–0.56), *p* = 0.009] were higher in the TAIL group. However, PG levels at visit 4 [0.42 (0.26–0.71) vs. 0.84 (0.63–1.34), *p* < 0.001] were lower (Figure 3).

In the TAIL group, the distribution of ovarian stimulation duration was as follows: 14 donors (20.59%) required 7 days, 19 donors (27.94%) required 8 days, 27 donors (39.71%) required 9 days, 3 donors (4.41%) required 10 days, 1 donor (1.47%) required 11 days, and 4 donors (5.88%) required 12 days of Controlled Ovarian Stimulation (COS). Notably, within this group, our analysis revealed a negative correlation between the duration of ovarian stimulation and oocyte collection outcomes, with longer cycles associated with poorer outcomes.

Out of a total of 21 donors (30.89%) in the TAIL group who had a low number of MII oocytes (<6 MII), seven donors (10.3%) had MII = 0. Among those with MII = 0, the triggering day for retrieval was as follows: one donor (14.28%) at 8th day, three donors (42.86%) at 9th day, and three donors (42.86%) at 12th day of COS (Table 4).

**Table 4:** MII number ≥6 contingency table and COS duration in TAIL group.

		COS duration in TAIL group (days)						Total
		7	8	9	10	11	12	
MII number	MII ≥6	12 (85.7%)	14 (73.7%)	19 (70.4%)	1 (33.3%)	1 (100%)	0 (0%)	47 (69.11%)
	0 < MII < 6	2 (14.3%)	4 (26.3%)	5 (29.6%)	2 (66.7%)	0 (0%)	1 (25%)	14 (20.59%)
	MII = 0	0 (0%)	1 (5.3%)	3 (11.1%)	0 (0%)	0 (0%)	3 (75%)	7 (10.30%)
Total		14 (20.59%)	19 (27,94%)	27 (39,71%)	3 (4,41%)	1 (1.47%)	4 (5,88%)	68 (100%)

COS, Controlled ovarian stimulation (days); MII, Metaphase II oocytes.

**Figure 4:**
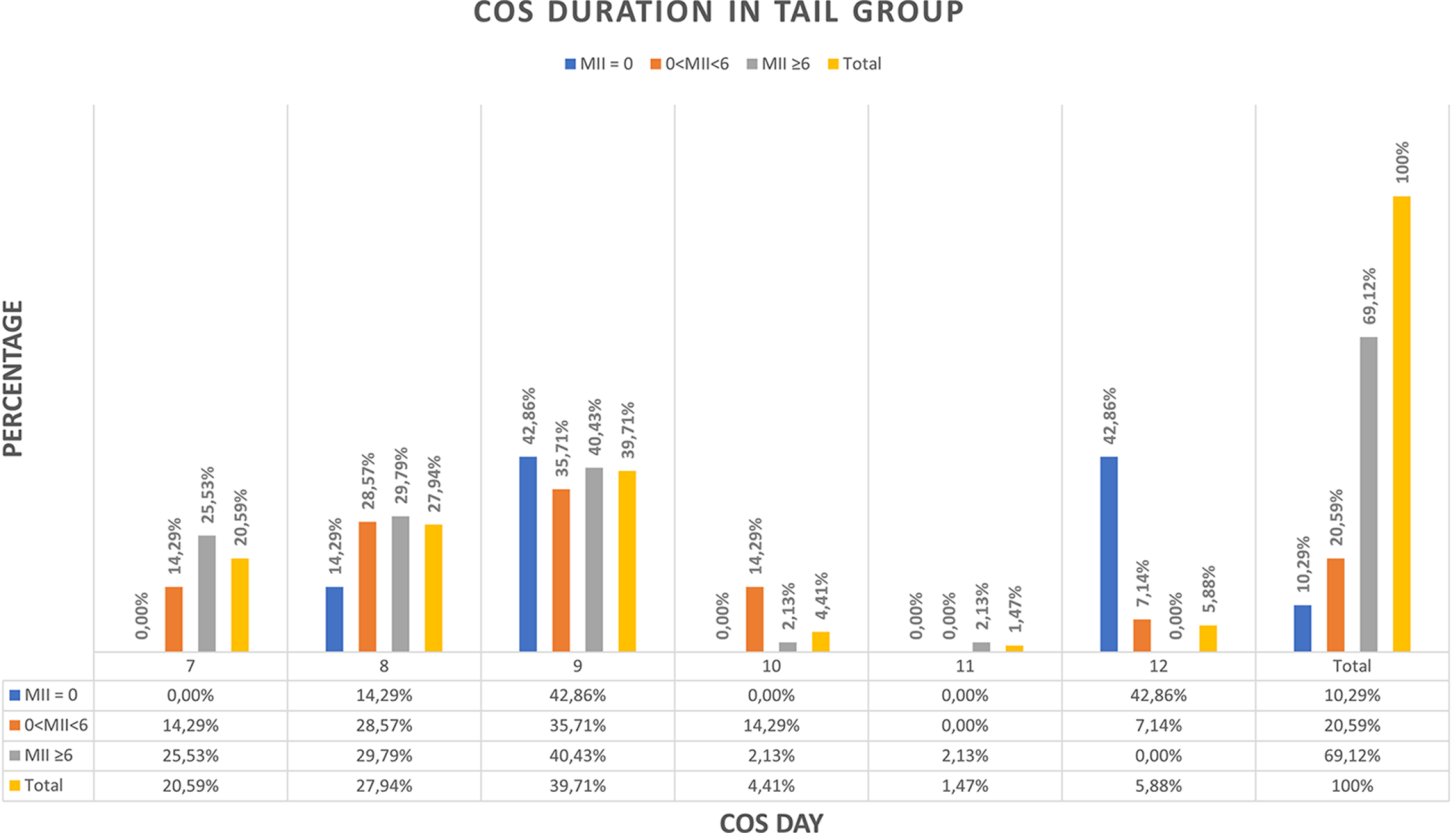
COS duration in TAIL group. Values are expressed as percentage. COS, Controlled ovarian stimulation; MII, Metaphase II oocytes.

Furthermore, when examining the relationship between the duration of COS and the percentage of oocyte retrievals with a low number of MII oocytes (<6 MII), we found that 100% of retrievals fell into this category if 12 days of COS were required, 66.7% for 10 days, 40.7% for 9 days, 31.6% for 8 days, and 14.3% for 7 days of COS. Notably, no MII oocytes were obtained at retrieval in 5.3%, 11.1%, and 75% of cases when 8, 9, and 12 days of ovarian stimulation were needed, respectively (Figure 4).

The total cost of gonadotropins in euros was significantly lower in the TAIL group compared to the control group [609.77 vs. 812.27 (756.02–913.52), *p* < 0.001]. However, the cost per MII oocyte collected (MII/€) was similar between the two groups [0.01 (0.01–0.02) vs. 0.01 (0.01–0.02), *p* = 0.65 for TAIL vs. control, respectively].

Furthermore, there were no statistically significant differences in the number of follicles larger than 14 mm and 17 mm at the time of triggering between the two groups (Table 2). Importantly, no drug-related complications or severe Ovarian Hyperstimulation Syndrome (OHSS) were observed during the study.

## 4. Discussion

Numerous studies, including ENGAGE (8), ENSURE (9), PURSUE (10) and others, have demonstrated that Corifollitropin alfa (CFA) is as effective as daily rFSH over a one-week period. These studies have shown equivalence in terms of stimulation duration, the number of retrieved oocytes (10–12), the number of mature MII oocytes, fertilization rates, pregnancy rates, incidences of Ovarian Hyperstimulation Syndrome (OHSS), and live birth rates (13, 14). This approach not only enhances donor compliance but also reduces the treatment burden (3, 15).

Our current study represents the first attempt to assess the efficiency of ovarian stimulation using a single 150 mcg injection of CFA without additional rFSH supplementation after a 7-day OCP-free interval, compared to the standard CFA protocol (5-day OCP-free interval) in oocyte donors. In this trial, the primary endpoint—the number of cumulus-oocyte complexes (COCs) and metaphase II (MII) oocytes following retrieval—was significantly lower in the TAIL group, with a difference of medians of 3.5 for COCs and 3 for MII oocytes. Rather than the hypothesized step-down-like effect, this unfavorable outcome appears to be associated with a coasting effect, indicated by lower serum FSH levels on the triggering day. Serum hormonal levels did not show differences in FSH levels at baseline, day 6, and day 8 of stimulation. However, the lower FSH level in the TAIL group on the triggering day suggests a reduced threshold for follicular stimulation starting from day 8 of stimulation.

Furthermore, subgroup analysis revealed an increased risk within the TAIL group for cycles collecting fewer than 6 MII oocytes or none (MII = 0), correlating with the number of days without rFSH supplementation. This negative effect became apparent as early as day 8 of Controlled Ovarian Stimulation (COS). While these results strongly suggest a coasting component explaining the adverse outcomes, they are intriguing in light of findings by Nardo et al. (16), reporting that only women coasting for 4 days had significantly fewer oocytes retrieved and a decreased implantation rate compared to those coasting for 1–3 days. Differences in pharmacodynamics between FSH and CFA and variations in the patient population may partially explain these discrepancies. On the contrary, 14 (20.59%) oocyte donors in the TAIL group completed COS in just 7 days, with no cases of MII = 0. This finding aligns with the results of Pérez-Calvo et al. (7) and the ENSURE Group´s findings (9) which showed no rFSH supplementation in about 12% to 32% of patients employing a GnRH antagonist protocol plus Corifollitropin alfa.

As anticipated, levels of estradiol (E2) and luteinizing hormone (LH) at baseline and on the 5th day of stimulation were higher in the TAIL group, reflecting the impact of the OCP-free interval. Cédrin-Durnerin *et al*. (17) conducted a study in which they measured and compared FSH, LH, E2, and progesterone levels after discontinuing OCP at 1, 3, and 5 days of pill-free intervals. They observed an increase in estrogen and LH levels as the OCP-free interval extended, indicating the release of the suppressive effects of the contraceptive pill on the pituitary gland. At baseline, on the 5th day, and on the 8th day of stimulation, E2 and LH levels remained elevated. In contrast, E2 levels on the triggering day were significantly lower in the TAIL group, providing further evidence of a coasting effect from the 8th day of Controlled Ovarian Stimulation (COS) onward. Huang *et al*. (4) conducted a study measuring E2, progesterone (PG), and LH levels in a standard protocol, and their results were consistent with those of our control group.

In our current study, we observed significant reductions in the duration of Controlled Ovarian Stimulation (COS) and the use of antagonist in the TAIL group, with differences of 0.54 and 0.58 days, respectively, compared to the control group. These findings are consistent with the results reported by Pérez-Calvo *et al*. (7) who also demonstrated enhanced efficiency when initiating COS after CFA administration following a 7-day OCP-free interval, in contrast to the same protocol with only a 5-day OCP-free interval. Their study revealed a shorter stimulation duration (9.3 [1.6] vs. 10.3 [1.6] days) and a reduced total additional dose of daily gonadotrophins (459 [356] vs. 659 [452] IU were required, respectively). However, considering that the donors in our study are typically young and likely to be long-term users of oral contraceptives, and given the random design of the study, it is reasonable to assume that these characteristics were evenly distributed between the two groups. Therefore, they are unlikely to have acted as confounding variables in our final results.

Additionally, our data revealed a lower total cost of gonadotropins in the TAIL group, as this group only required a single medication, while the control group needed an additional of 3 (2–3) days of 225 IU of rFSH. However, it's essential to note that the TAIL group's lower oocyte yield and maturation rate significantly impact its cost-effectiveness as a Controlled Ovarian Stimulation (COS) protocol.

Regarding other endpoints, such as the number of follicles larger than 14 mm on the day of the agonist trigger and the cost per mature oocyte collected (in euros), no statistically significant differences were observed between the two groups.

It's worth mentioning that our study did not initially intend to analyze pregnancy or fertilization rates in the matched recipients, and this analysis was not performed due to the storage of numerous oocytes. This underscores the importance of future research endeavors in gathering comprehensive data, which should include an assessment of other key metrics such as blastocyst formation rates, and clinical outcomes including the incidence of live births.

## 5. Conclusions

Although a shorter duration of Controlled Ovarian Stimulation (COS) was observed in the TAIL group, likely linked to a reduced suppressive effect of the contraceptive pill after a 7-day pill-free interval, our study also revealed a significant decrease in the number of mature oocytes (MII) and cumulus-oocyte complexes, as well as lower hormone levels (FSH and E2) on the day of trigger in the TAIL group. These findings appear to be associated with a coasting effect. In summary, our data suggests that the use of a single administration of Corifollitropin alfa (CFA) after a 7-day pill-free interval, without additional FSH supplementation after the first seven days, may not maintain the threshold level required for optimal follicular stimulation.

## Data Availability

The datasets presented in this study can be found in online repositories. The names of the repository/repositories and accession number(s) can be found below: https://www.clinicaltrialsregister.eu/ctr-search/trial/2019-001343-44/results.
